# The Recent Progress of the Cellulose-Based Antibacterial Hydrogel

**DOI:** 10.3390/gels10020109

**Published:** 2024-01-29

**Authors:** Ying Sun, Jiayi Wang, Duanxin Li, Feng Cheng

**Affiliations:** 1 College of Light Industry and Textile, Qiqihar University, Qiqihar 161006, China; wjy5266@sina.com (J.W.); ldx0711@sina.com (D.L.); 2 Cold Area Hemp and Products Engineering Research Center of Ministry of Education, Qiqihar 161006, China; 3 School of Chemistry and Chemical Engineering, Harbin Institute of Technology, Harbin 150001, China

**Keywords:** antibacterial materials, cellulose-based, hydrogels, cross-linking preparation, application

## Abstract

Cellulose-based antibacterial hydrogel has good biocompatibility, antibacterial performance, biodegradability, and other characteristics. It can be very compatible with human tissues and degradation, while its good water absorption and moisturizing properties can effectively absorb wound exudates, keep the wound moist, and promote wound healing. In this paper, the structural properties, and physical and chemical cross-linking preparation methods of cellulose-based antibacterial hydrogels were discussed in detail, and the application of cellulose-based hydrogels in the antibacterial field was deeply studied. In general, cellulose-based antibacterial hydrogels, as a new type of biomaterial, have shown good potential in antimicrobial properties and have been widely used. However, there are still some challenges, such as optimizing the preparation process and performance parameters, improving the antibacterial and physical properties, broadening the application range, and evaluating safety. However, with the deepening of research and technological progress, it is believed that cellulose-based antibacterial hydrogels will be applied and developed in more fields in the future.

## 1. Introduction

Diseases caused by bacterial infections have seriously threatened human health, and the longterm overuse of antibiotics can easily lead to bacterial resistance, resulting in the emergence of multidrug-resistant bacteria [1,2]. With the widespread use of antibiotics and other drugs, infections caused by pathogenic micro-organisms have been alleviated to a certain extent. However, the overuse and improper use of these drugs have led to an increase in the resistance of pathogenic micro-organisms, and wound infections are also one of the most common phenomena in the medical field [3]. At present, some wound dressings may contain glia, which can cause skin irritation or allergies. Due to the limited absorption capacity, dressings may not be able to fully absorb a large amount of exudate or large wounds, leading to wound infection or attachment detachment. Therefore, the development of antibacterial materials to improve the problem of infection has been urgent [4].

Cellulose has a large number of pores and micropores, which can adsorb a large amount of water and other substances and has high crystallinity. It exhibits stable performance in high-temperature environments and is not easily decomposed or deformed. 

Cellulose is biodegradable and can be broken down into water and carbon dioxide under the action of oxygen and micro-organisms, making it an environmentally friendly biomaterial. At the same time, there are multiple active functional groups in cellulose that can participate in chemical reactions, carry out modification grafting, and improve the hydrophilicity and biological activity of cellulose. Cellulose has become an ideal wound dressing with high practical prospects due to these advantages. For example, cellulose antibacterial hydrogels can achieve effective antibacterial treatment by controlling drug release. The preparation method of this hydrogel is to use the hydrophobicity and cross-linking reaction of natural cellulose to wrap the drug in the hydrogel network. In the body, drugs can be slowly released to achieve sustained antibacterial effects. By changing the cross-linking degree and drug content of cellulose, the release rate of drugs can be regulated to meet different treatment needs. Another kind of cellulose antibacterial hydrogel was developed for wound hemostasis. This hydrogel coagulates cellulose, platelets, and coagulation factors through electrostatic action to prevent thrombosis. Due to the natural porosity of cellulose, hydrogels can effectively absorb water and ions in the blood, and promote platelet aggregation and the activation of coagulation factors, and, thus, quickly stop bleeding. At the same time, the hydrogel also has antibacterial properties, which can effectively prevent infection. Cellulose antibacterial hydrogels can also be used to promote wound repair. One preparation method is to modify cellulose using biocompatible materials to enhance its biocompatibility and antibacterial properties. This kind of hydrogel can be used as a protective layer of the wound surface, provide a humid environment, and promote cell proliferation and migration. At the same time, the antibacterial ingredients in the hydrogel can effectively prevent infection and promote wound healing. By adjusting the molecular weight of cellulose and the type of biocompatible materials, the performance of the hydrogel can be optimized to achieve the best wound repair effect. 

Cellulose-based antibacterial hydrogels, as one of the important types of macromolecular antibacterial agents, have the functions of being antipollution, anti-secondary trauma, and promoting cell regeneration.

In addition, they also have the dual functions of being a hydrogel and antibacterial, strong biocompatibility, strong water absorption, controllable mechanical properties, and other advantages. At present, cellulose-based hydrogels have received extensive attention and have become a hotspot in research and development [5]. It plays an important role in many fields such as tissue engineering, drug release, injectable drugs, wound dressings, etc. [6]. However, currently, commonly used antibacterial hydrogels often have disadvantages such as poor heat resistance, difficult large-scale production, large metal toxicity, and a bactericidal effect only in the presence of ultraviolet radiation and oxygen (or water) [7,8]. Therefore, the new antibacterial hydrogels with the properties of being convenient, non-toxic or low-toxicity, effective, stable, not easy to produce drug resistance, and good biocompatibility have been the research focus of experts and scholars.

## 2. Structure and Properties of Cellulose

Cellulose is one of the natural biopolymers with abundant sources and wide distribution. It is naturally synthesized and is considered a very valuable raw material developed from renewable resources. It has the characteristics of non-toxicity, universality, biodegradability, and good compatibility [9,10], making cellulose and its derivatives potential materials for addressing industrial and environmental challenges. Due to the natural and renewable advantages of cellulose, whether it’s used in simple daily necessities or complex industrial products, cellulose has a wide range of applications, such as plastics, paperboard, fibers, textiles, cellulose derivatives, and a lot of complex materials [11]. Cellulose exists in plants, animals, germ, phycophyte, and fungus, and is mainly derived from plant fibers, such as wood, bamboo, cotton, hemp, etc. The main component of the plant cell wall is cellulose, and this cell wall has the characteristics of resistance to tension strength and rigidity without deformation [12].

**Figure 1:**
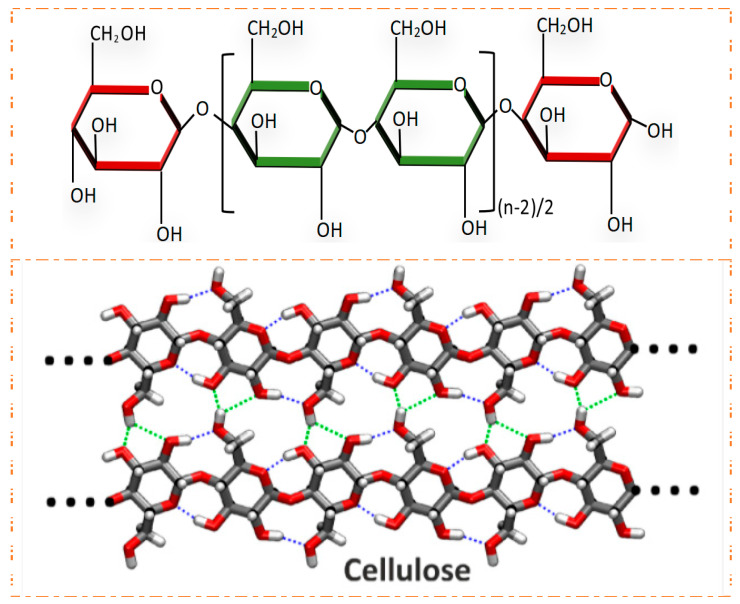
Cellulose structure formula. Reproduced with permission [13].

Cellulose is a linear, stereotactic, semi-crystalline polysaccharide [13], with a unique three-dimensional cross-linked porous structure and rich functional groups (Structural formula of cellulose. Figure 1). It is made up of repeated monomer D-glucose [AGU], which is successively connected by β-1,4 bonds between C1 and C4 of adjacent units [14] to form strong intermolecular hydrogen bonds, resulting in crystallized and amorphous areas, and forming microfilaments and fibers [15]. Long cellulose chains are horizontally connected together to form basic nanofibers and their bundles, called microfibers, which contain microcrystals and amorphous domains. Cellulose microcrystals cannot be in contact with water, while amorphous regions are easily in contact with water [16]. Many cellulose products, such as cellulose acetate and carboxymethyl cellulose, can be obtained by a surface modification of cellulose. Its structure includes crystalline and partially amorphous regions, and smaller cellulose nanofibers and cellulose nanocrystals can be obtained by mechanical force treatment or chemical acid and enzyme treatment.

## 3. Cellulose-Based Antibacterial Hydrogel

Cellulose-based antibacterial hydrogels were prepared by dissolving, blending, and cross-linking using cellulose, nanocellulose, or cellulose derivatives as main raw materials. Cellulose contains a large number of hydroxyl groups, which is conducive to the interaction between molecules and other substances to form cross-linking and form a gel network. Cellulose itself is insoluble in water and does not have antibacterial properties, so it can be modified to improve the solubility and antibacterial properties of modified cellulose [17]. 

**Figure 2:**
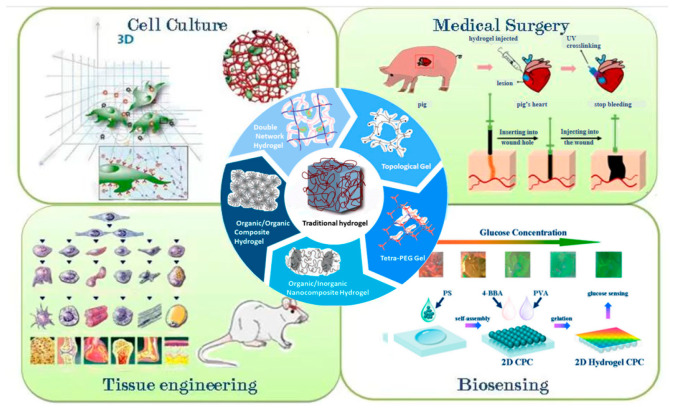
The middle part is the application of traditional hydrogels, while the remaining four parts are the application of cell culture, medical surgery, tissue engineering, and biosensing. Reproduced with permission [18].

The excellent properties of hydrogels, such as hydrophilicity and porosity, can be developed for antimicrobial applications. Its various production methods have been widely applied in fields such as biomedical science and smart textiles, for example, cell culture, medical surgery, tissue engineering, biosensing (Figure 2), etc. [18], and it is one of the biomaterials suitable for drug sustained release in the field of antimicrobial therapy [19]. The effect of traditional hydrogels on bacteria is mainly due to the presence of antibiotics or other antimicrobial substances in the hydrogel substrate, as well as the physical and chemical properties of the hydrogel itself. However, the increase of other antibacterial substances not only increases the composite method and cost of hydrogels, but also may lead to a discrepancy in the release of antibacterial substances, showing inconsistent antibacterial activity. As an antibacterial product, hydrogels can perform multiple functions at the same time, and the most important properties are antibacterial. The bacterial resistance properties of antibacterial hydrogels are determined by the antibacterial components, and the antimicrobial properties of different materials also vary greatly, especially for different strains [20]. Antibacterial hydrogels have attracted much attention due to their simple preparation process, diverse structure, wide variety, and the fact that they are highly anticipated [21]. 

Compared with other antibacterial materials, the antibacterial hydrogel has many excellent properties: (1) The production cost of this antibacterial product is low and its preparation is convenient [22,23,24]. For example, the photosensitive antibacterial composite hydrogel can be prepared by a controlled method, which can obtain different antibacterial activities and mechanical strength [25,26,27]. The hydrogel with a higher antibacterial activity can be prepared by adjusting the process parameters [28], which can avoid a drug fast by rapidly dispatching bacteria [29]. (2) The antimicrobial hydrogel administration method is simple. They have good external adhesion and can adhere to the surface of damaged instruments and tissues [30]. Antibacterial hydrogels also have excellent injectable properties and can be used for internal treatment. This means in vivo application. They are injectable into blocked areas of the skin using needle injection instruments and make use of minimally invasive methods for treatment [31]. In addition, compared with ordinary medical antibacterial dressings, antibacterial hydrogels reduce wound infection to a certain extent and speed up wound healing [32]. (3) Hydrogel is an excellent biological carrier. In terms of composition, basic structure, and functional characteristics, hydrogels are similar to some tissues in the human body. As a consequence, they have excellent biocompatibility and biodegradability, and can maintain or control the hydrogel entering the body and releasing [33,34]. At the same time, hydrogel also plays a carrier role for drugs, which can slow down the stimulation on the human body to a certain extent [35,36]. 

**Figure 3:**
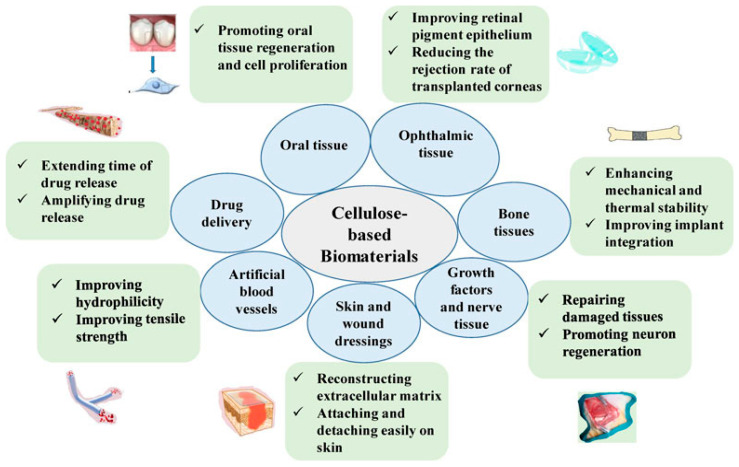
Biomedical applications and advantages of cellulose-based materials. Reproduced with permission [38].

Cellulose and its derivatives can form hydrogels, films, aerogels, and other materials with various structures. Its rich pore structure and large surface area make it very convenient for mineralization. Cellulose has great potential in the preparation of bio-based superabsorbent hydrogels. At the same time, the multi-layer structure and abundant hydroxyl groups of cellulose enable it to enhance its functionality through chemical modification. Cellulose and its derivatives can be used as substrates to deposit different types of inorganic minerals, forming multifunctional organic/inorganic composite materials. From the perspectives of socio-economic, ecological, and environmental protection, physical and chemical properties, and biology (Figure 3), cellulose is preferable for the production of hydrogels. It has attracted great attention because it is safe, biodegradable, biocompatible, non-toxic, non-immunogenic, and relatively inexpensive; has strong mechanical properties, high thermal stability, special porous structure, and good biocompatibility; and is suitable for human consumption [37]. Owing to the common disadvantage of poor solubility of natural cellulose, many chemical operations are often required in the process of preparing hydrogels from cellulose derivatives, but there are many techniques for preparing hydrogels from non-cellulose derivatives, which can be dissolved and regenerated from multiple sources. The abundant hydrophilic functional groups (hydroxyl groups) in the chemical structure of cellulose make it an ideal raw material for hydrogel preparation.

Cellulose-based antibacterial hydrogel is a kind of polymer material with a three-dimensional network structure and hydrophilic chain. They are prepared by physical and chemical cross-linking or interpenetrating polymer network methods using cellulose and its derivatives as the main raw materials and aqueous solution as the dispersion medium [39,40]. Due to its unique polyhydroxyl network structure and high degree of polymerization of rigid molecular chains, it has good potential in forming hydrogels with excellent mechanical properties. Generally, cellulose is dissolved in solution and easily forms hydrogels through the hydrogen bond network structure induced by poor solvents or through chemical cross-linking by adding cross-linking agents. In this case, there is no need to use synthetic cellulose derivatives, and the ability of hydroxyl groups to form hydrogen bonds (self-assembly) or the reaction between hydroxyl groups and cross-linking agents are mainly utilized. This pure cellulose hydrogel retains the original hydrogen bond network, which makes it stronger.

At present, the preparation of cellulose antibacterial hydrogels usually includes the following ways: (1) Cross-linking after direct dissolution. However, cellulose is prone to forming hydrogen bonds between the hydroxyl groups, resulting in a high degree of crystallization, so cellulose is difficult to dissolve in water and common solvents [41]. (2) Cross-linking after derivatization and dissolution. The original crystalline structure of cellulose can be destroyed by chemical modification, and cellulose derivatives can be formed to dissolve cellulose. (3) Small-size stable dispersion in the solvent after cross-linking. This preparation method is most commonly used for nanocellulose, which is a good way to avoid the problem of cellulose dissolution.

## 4. Cross-Linking Preparation Method of Cellulose-Based Hydrogel

### 4.1. Preparation Methods of Physically and Chemically Cross-Linked Hydrogel

**Figure 4:**
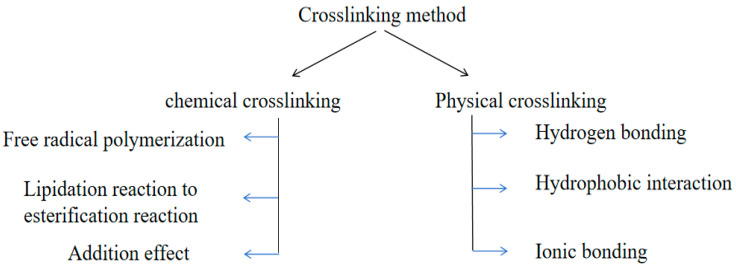
Cross-linking method of cellulose-based antibacterial hydrogels.

Cellulose-based antibacterial hydrogels are formed by physical or chemical cross-linking between molecular chains in the system (Figure 4). Cross-linking not only makes cellulose chain structures interact to form three-dimensional structures, but also improves the physical and mechanical properties of hydrogels.

### 4.2. Preparation of Physical Cross-Linking Hydrogels

Physical cross-linked hydrogels are three-dimensional network structures formed by non-chemical bonds such as hydrogen bonds, ionic bonds, and van der Waals interactions [42,43]. The reversible nature of this action leads to the structural instability of the corresponding hydrogels, but, at the same time, gives the hydrogels self-healing properties.

#### 4.2.1. Hydrogen Bonding

Individual hydrogen bonds are weak, but, when the system contains a large number of hydrogen bonds, the synergistic effect can provide sufficient stability for the hydrogel. Cellulose molecular chains are rich in hydroxyl groups, which makes it possible for cellulose to construct hydrogel networks through hydrogen bonding. The properties of cellulose gels are related to the concentration, polymerization degree, and viscosity of cellulose. Liu [44] et al. prepared the cellulose gel membrane by a curing and regeneration process in the solvent system of 1-ethyl-3-methylimidazolium aminoacetate. In ionic liquid solvent systems, cellulose/polycaprolactone blend films are prepared by hydrogen bonding.

#### 4.2.2. Hydrophobic Interaction

In the process of hydrogel cross-linking, hydrophobic interactions exist between water-soluble polymers and hydrophobic end groups, side chains, or monomers, which can drive hydrophobic groups or non-polar molecules to gather together to form a network structure. When the hydroxyl group of cellulose is partially replaced by the methyl group, the formation of hydrogen bonds between cellulose molecular chains is often restricted, resulting in water-soluble cellulose derivatives [45]. Some water-soluble cellulose derivatives exhibit unique thermal reversible gelation properties under hydrophobic interaction; that is, hydrogels can be formed within a certain temperature range. But this hydrogel is reversible; when the temperature changes, it will return to the sol state. Jung et al. [46] adopted β-chitosan nanocrystals as fillers to promote gelation through polarity and hydrophobic interactions, thereby improving the mechanical strength of hydrogels.

#### 4.2.3. Ionic Bonding

The charged polymer and the oppositely charged ions form a physical hydrogel through ionic bonding, which is called an “ionic” hydrogel. Werner et al. combined negatively charged cellulose sulfate with chitosan and synthetic cationic polyelectrolyte, and prepared biocompatible hydrogels that can be used in the biomedical field [47,48]. Lu [49] et al. studied the solgel transition process in the mixed system of the positively charged quaternary ammonium hydroxyethyl cellulose ethoxyl compound and negatively charged nanocellulost, and successfully determined the gel point. Hussain et al. [50] designed a supramolecular hydrogel composed of polyacrylamide and hydroxyethyl cellulose with self-healing properties by hydrogen bonding and cross-linking with Fe^3+^. The structural stability and mechanical strength of hydrogels depend on the type, concentration, density, and cross-linking degree of the polymer used.

### 4.3. Chemical Cross-Linking

Chemical hydrogels are formed by covalent bonds, and this cross-linking is irreversible, so the mechanical properties of corresponding hydrogels are usually better than those of physical hydrogels. 

#### 4.3.1. Free Radical Polymerization

The presence of a large number of hydroxyl groups in the molecular chain of cellulose makes it easy to be modified by free radical polymerization [51]. In the process of free radical polymerization, the initiator induces the generation of free radicals, resulting in the formation of active sites on the cellulose chain and the formation of a hydrogel skeleton, which provides necessary conditions for subsequent binding with monomers [52]. Then, the monomer or cross-linking agent combines with the active site on the cellulose chain and promotes the expansion of the polymer chain, thus forming a hydrogel network. The properties of hydrogels formed by free radical polymerization are affected by the initiator, cross-linking agent, reaction time, and temperature. Maity et al. [53] prepared hydrogels that could adsorb copper by polymerizing acrylamide or acrylic monomer with bagasse cellulose and gelatin through the polymerization of a free radical. The polyacrylamide glycolic acid/cellulose nanocrystalline hydrogel synthesized by Rao et al. [54] can be used as a carrier for oral drug release.

#### 4.3.2. Esterification Reaction

The hydroxyl group on cellulose and the hydroxyl group and carboxyl group contained in its derivatives make it possible to prepare cellulose-based hydrogels by esterification. Kono et al. [55] prepared superabsorbent cellulose hydrogels by esterifying and cross linking 1, 2, 3, and 4-butane tricarboxylic acid dianhydride in a lithium chloride/n-methyl-2-pyrrolidone solvent system. Demitri et al. [56] prepared carboxymethyl cellulose/hydroxyethyl cellulose hydrogel by esterification using carboxymethyl cellulose (CMC) and hydroxyethyl cellulose (HEC) as raw materials and sodium citrate (CA) as the cross-linking agent. Because the raw material used is renewable and easily degradable, the hydrogel can be used in the agriculture and biomedicine fields. Senna et al., using cellulose acetate (DS) as the raw material and catalyzed by triethylamine, cross-linked with ethylenediamine tetraacetic anhydride (EDTAD), prepared hydrogels which can be used as soil water retaining agents [57].

#### 4.3.3. Addition Effect

All substances containing double bonds can be cross-linked with hydroxyl groups on cellulose and its derivatives by an addition reaction to prepare cellulose-based hydrogels. Esposito et al. [58] prepared a highly absorbent material using sodium carboxymethyl cellulose and hydroxyethyl cellulose as raw materials and divinylsulfone as the cross-linking agent. Butun [59] prepared temperaturesensitive HPC microgels and magnetically responsive carboxymethyl cellulose hydrogels using divinyl sulfone as cross-linking agent. 

### 4.4. Double Cross-Linked Hydrogels

The traditional chemical cross-linked mononet hydrogel can only construct a three-dimensional network through covalent bond cross-linking in the system due to its single structure, so it cannot bear great strain, which greatly restricts the development and application of hydrogel. In order to improve the mechanical properties of hydrogels through cross-linking mechanisms, researchers have pioneered a chemical physical double cross-linking method, in which two different functional groups form two different cross-linking points in a single network of hydrogels. The hydrogels with different strengths can be obtained by adjusting the amount of cross-linker while improving the mechanical strength of hydrogels. 

## 5. Application of Cellulose-Based Hydrogels in the Field of Antibacterial

### 5.1. Cellulose-Based Antibacterial Hydrogels Containing Metal Ion Materials

In recent decades, the overuse and misapplication of antibiotics have posed serious challenges in the field of healthcare [60]. Therefore, we are in need of a new succedaneum for antibiotics. The development of nanotechnology has provided many new ideas and methods for the development of new antibacterial materials. The usual inorganic antibacterial materials mainly include metal ions and metal oxide nanoparticles. Metal ions mainly include silver ions (Ag^+^) and copper ions (Cu^2+^) [61,62,63,64,65], which can combine with bacterial cell membranes through mercaptan groups on cell membranes and affect their activity by controlling the copy of bacterial DNA [66,67,68,69]. Nevertheless, metal nanoparticles are prone to aggregation, dissociation, and combustion, which greatly limit the application of antibacterial drugs [70]. Cellulose-based antibacterial hydrogels are excellent supporters for wearing metal nanoparticles [71]. Due to the slow release properties, they are an appropriate footplate for carrying metal nanoparticles. Metal nanoparticles can be valuably decorated in cellulose hydrogels and gradually released, which will bring persistent antibacterial effects to the hydrogels [13]. 

Bundjaja et al. [72] researched the influence of surfactants on the antibacterial activity of the cellulose carbamate hydrogels of silver nanoparticles by adopting cellulose carbamate hydrogels loaded with silver nanoparticles. A new method of introducing silver nanoparticles into hydrogels was introduced. The silver ions were transferred to the surface active sites of hydrogels by silver ion adsorption technology, and then the silver ions were reduced in situ. Then, the cellulose carbamate hydrogel was modified with the surfactant to obtain cellulose carbamate brine gel loaded with AgNPs. Finally, we measure the concentration of embedded silver ions using an atomic absorption spectrophotometer. This method enables a large amount of AgNP to be bound to the surface of the hydrogel dressing, thereby enhancing the antibacterial activity against the target bacteria. In addition, this method may be an improvement on the traditional AgNPs hydrogel dressing. Therefore, because it is difficult to reach the target bacteria, the effectiveness of AgNPs in the center of the hydrogel matrix is reduced. Finally, the antibacterial activity of the hydrogel samples was determined by the paper diffusion method. We test the inhibitory effect of the sample on two bacterial strains, namely, Escherichia coli and Staphylococcus aureus. The experiment shows that the addition of the surfactant can effectively improve the loading of AgNPs on hydrogel dressings and promote antibacterial activity. In addition, a cytotoxicity test showed that the hydrogel dressing had a good biocompatibility with skin fibroblasts.

Al-enizi et al. [73] researched cellulosehydrogels-animated CuNPs by the polymer cross-linking method. CMC-based hydrogels were prepared with PVA and EGDE in aqueous solution. Copper ions are bound to the hydrogel matrix by electrostatic interaction with the oxygen atoms of the hydrogel. Antibacterial activity and cytotoxicity studies were also conducted on urinary tract infection micro-organisms and HeLa cells, respectively. The results showed that the HCuNPs composite had a higher antibacterial zone than the corresponding hydrogel matrix, and the prepared nanocomposite could be used as a promising candidate for biomedical applications. The composite hydrogel had a good antibacterial effect and good biocompatibility. Therefore, due to the water absorption, antibacterial properties, and biocompatibility of hydrogels, hydrogels have the potential to be developed for women’s sanitary napkins, diapers, and other related care products. 

### 5.2. Cellulose-Based Antibacterial Hydrogels Containing Metal Oxide Materials

Similar to metal nanoparticles, cellulose-based hydrogels containing metal oxide nanoparticles can effectively kill bacteria, reduce drug resistance, and improve antibacterial properties. It is more difficult for metal oxide nanoparticles to release metal ions; metal oxide nanoparticles are also easy to agglomerate. The long-term biological virulence and environmental accumulate virulence problems need to be further defined and resolved. These shortcomings will weaken its antibacterial effect, so we need to develop new modification methods to overcome these shortcomings. Different from the antibacterial mechanism of metal nanoparticles, metal oxide nanoparticles kill micro-organisms mainly through photocatalysis to produce a large number of free radicals [74]. Among various metal oxides, zinc oxide (ZnO) is the most commonly used antibacterial agent [75], which provides outstanding antibacterial functions for cellulose hydrogels and improves the thickness of hydrogel net. The dense hydrogel network further enhances the result of hydrogel shipping and slowly releases the antiseptic, thus extending the antibacterial time-efficiency of hydrogels [76]. In addition, the chemical stability and antioxidant ability of metal oxide nanoparticles are one of the important reasons for their excellent performance in various application fields [77,78].

**Figure 5:**
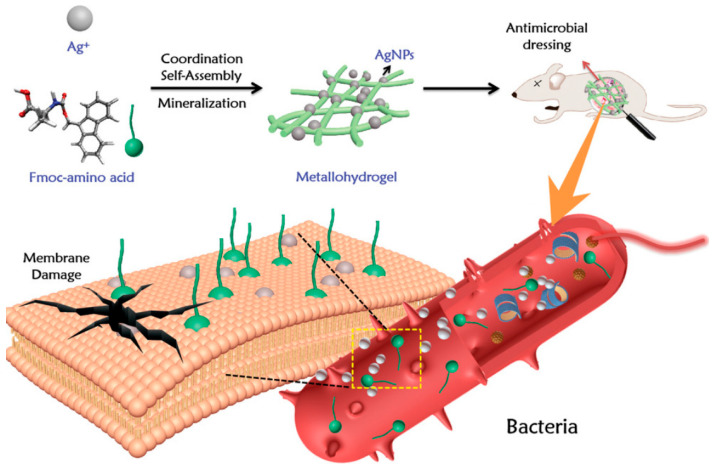
A biometallic hydrogel process based on Ag-Fmoc-amino acid co-ordination complex, which generates AgNPs precursors through mild mineralization process and self-assembles with broad-spectrum antibacterial activity and adjustable mechanical properties. Reproduced with permission [82].

Yuan et al. reported the self-assembly of broad-spectrum antibacterial metal hydrogels based on the Ag^+^ co-ordination of FMOC-amino acids (Figure 5) [79]. Biometallic hydrogels have broad-spectrum antibacterial activity and adjustable mechanical properties, based on the co-ordination of the self-assembly of biocompatible amino acids and silver ions. The high directionality of the co-ordination interaction makes Ag^+^ immobilized in the amino acid nanofiber hydrogel. As an antibacterial material in vivo, this metal hydrogel has the characteristics of having a simple and flexible strategy, and good biocompatibility and biodegradability. The metal hydrogel showed strong antibacterial activity and improved the in vivo therapeutic efficacy in infected rat models. Yadollahi et al. [80] prepared the composite hydrogel by synthesizing ZnONPs in situ in the expanded carboxymethyl cellulose hydrogel. Due to the porous structure of the hydrogel and the presence of carboxylic acid groups, the hydrogel is easy to combine with zinc, and cations in the zinc nitrate aqueous solution interact through the co-ordination interaction. Under the action of appropriate alkaline reagents such as NaOH, zinc ions become ZnO nanoparticles. The experiment proved that the nanocomposite hydrogel showed the antibacterial effect against Escherichia coli and Staphylococcus aureus, and the antibacterial activity increased with the increase of the concentration of ZnONPs in the hydrogel. George et al. [81] used cross-linked dialdehyde cellulose prepared from sugarcane cellulose and chitosan to prepare cellulose composite hydrogels containing ZnONPs, mixed them with ZnONPs synthesized from melon seed extracts, and applied them to the delivery of curcumin. Zinc oxide nanoparticles were synthesized from sweet melon seed extract and embedded in the hydrogel matrix, and cross-linked with dialdehyde cellulose prepared from sugarcane bagasse. The determination of antibacterial activity showed a synergistic antibacterial effect on Staphylococcus aureus and Trichophyton rubrum. It was found that the antibacterial activity of the hydrogel added with ZnONPs was significantly improved. 

### 5.3. Cellulose-Based Antibacterial Hydrogels Containing Antibiotics

Antibiotics have become the preferred treatment for bacterial contamination due to their efficient disinfection ability and low toxicity to mammalian cells [82]. Although antibiotics are the most common and effective antimicrobials, bacterial resistance, environmental fate, drug resistance, the short span of antibacterial activity, partly runaway concentrations, and degradation in some employ conditions [83,84,85] have been the biggest obstacles to antibiotic development and application. In order to solve this problem, we need to design and develop an efficient and biocompatible drug delivery system. This system should meet the strict requirements of low cytotoxicity while also exhibiting excellent antibacterial effects. Through such a system, we can effectively deliver drugs while reducing the toxic effects on cells, thereby more effectively treating infections [86].

Forrello-doria et al. [87] prepared drug-loaded composite hydrogels by using cross-linked cellulose and carbon nanotubes. The experimental results showed that the release rate of therapeutic substances from the hydrogel was improved, showing a simultaneous and co-ordinated release. Research on wound closure and in vivo wound healing has confirmed the effectiveness of the drug release and improved healing outcomes. Antibacterial experiments showed that linezolid had a very good sustained release effect, and the drug-loaded composite hydrogel prepared by this method had great potential to promote wound healing. Iman et al. prepared nanocomposite hydrogels containing polyethylene glycol, acrylamide, methylene bisacrylamide, and cellulose nanofibers [88], and tested the antibacterial activity of tetracycline nanocomposite hydrogels against Staphylococcus aureus and Escherichia coli. Nanocomposite hydrogel was used for an oral toxicity test in adult male Wistar rats. Studies on antibacterial activity have shown that the prepared compound is sensitive to Staphylococcus aureus and Escherichia coli. It was found that the hydrogel had good biocompatibility. Therefore, these formulations can be considered as future oral delivery systems. Patwa et al. [89] prepared magnetic cellulose composite hydrogels by using ionic cross-linking between alginate and casein and doping bacterial cellulose modified by magnetic nanoparticles. The antibacterial activity of the hydrogel was evaluated by an agar diffusion test of Escherichia coli and Staphylococcus aureus. The hydrogels were also characterized in detail, and it was observed that the interaction led to the expected properties of injectable hydrogels. The effects of different proportions of BCF on the morphology, swelling, magnetic, compression, and rheological properties of hydrogels were studied. The mouse embryonic fibroblasts were used to evaluate the cytocompatibility. The experimental results showed that the hydrogel had no cytotoxicity and was suitable for transdermal administration.

Today, antibiotics are still the first choice in the field of biomedicine to fight bacterial infections; due to the fatal shortcomings of antibiotics posing a serious threat to human health and hygiene safety, we need to pay attention to two aspects in the follow-up research of the joint application of antibiotics and cellulose hydrogels. On the one hand, we need to systematically develop new antibiotics with better antibacterial effects and lower drug resistance, which will help reduce antibiotic abuse and slow down the development of drug resistance. On the other hand, it is necessary to further explore the application potential of cellulose hydrogel as a drug carrier, and improve its combined application effect with antibiotics by optimizing its structure, performance, and biocompatibility, so as to provide a safer and more effective choice for antibacterial treatment.

### 5.4. Cellulose-Based Antibacterial Hydrogels Containing Biological Extracts

Biological extracts come from plants and animals. For quite some time, plant extracts, as an important component of new antibacterial agents, have always been a major research route for researchers at home and abroad, and the proportion of antibacterial ingredients extracted from plants in the medical field is increasing. Especially confronted with the critical terror of drug-resistant viruses, extracting antibacterial agents from plants shows a great capacity in treating drug-resistant viruses, and it is in line with human health and safety, efficiency, and environmental protection. Extracting and further developing new and efficient antibacterial compounds from plants has become a hot topic in research [90]. Ge et al. [91] used borax to cross-link cellulose nanofibers with PVA, and mixed TA with natural product TA to obtain TA-PVA cellulose composite hydrogel. By using cellulose nanofibers (CNFs) and tannic acid (TA) as functional additives, in the hydrogel, cellulose nanofibers and polyvinyl alcohol are connected by a dynamic borate ester bond and hydrogen bond. Due to the complexation of TA, unique antioxidant and antibacterial properties were obtained. It was found that the hydrogel had good antioxidant properties, high ductility, plasticity, and rapid self-healing advantages. The supported polyacrylamide hydrogels developed by Ravindra et al. show excellent antibacterial activity and have great therapeutic value [92]. In the research process of biological extraction in animal structures, bees are extremely easy to extract. Honey has shown efficient antibacterial performance in most wound-healing treatments [93,94]. Vitamin E (VitE) is a significant extract with antioxidant and biodegradable properties. VitE-functionalized polycarbonate hydrogel for antimicrobial applications has good compatibility with human cortical fiber cells. It can be loaded with cationic polymers at minimal bactericidal concentrations to kill bacteria and fungi [95]. With the increasing harm of drug-resistant viruses to the human body, plant-derived antibacterial components showed low resistance during the antibacterial process, and components extracted from plants have low resistance to antibiotics in the human body during the antibacterial process, bringing hope for fully addressing the damage of drug-resistant viruses. Furthermore, plant extracts are a rich and diverse treasure trove in nature, with extensive and easily accessible resources. The ingredients extracted from various plants are diverse and have low toxicity, making them friendly to humans and the environment; at the same time, the extraction process is relatively simple and convenient. More importantly, plant extracts have enormous potential to provide humans with a large number of efficient and highly resistant new antibacterial agents. By utilizing these natural antibacterial ingredients, one can better protect oneself and others from infection and reduce the dependence on traditional antibiotics. This not only helps to maintain human health, but also helps to protect our ecosystem. Therefore, in-depth research on the antibacterial mechanism and activity of plant extracts, as well as the preparation of new antibacterial agents, has profound significance and enormous research value. The combination of plant extract and cellulose hydrogel is an innovative method, which helps to dissolve the plant extract, stabilize the structure, and control the release, and this research direction has immeasurable value in promoting the application of plant extracts in the antibacterial field.

### 5.5. Cellulose-Based Antibacterial Hydrogels with Synergistic Effects

A synergistic hydrogel is one that contains two or more antimicrobials to enhance antibacterial action. Wang et al. constructed a copper-sulfide-nanoparticle-doped antibacterial peptide complex hydrogel to treat wound infection and promote wound healing [96]. Antibacterial peptide hydrogels can eradicate bacterial colonies or inhibit bacterial growth. CuSNDs can produce ROS and photothermal effects under near-infrared irradiation, showing excellent antibacterial activity. Nd-doped hydrogels effectively root out Staphylococcus aureus in infected wounds and promote rapid wound healing under NIR irradiation.

## 6. Application of Cellulose-Based Antibacterial Hydrogel

### 6.1. Wound Dressings

**Figure 6:**
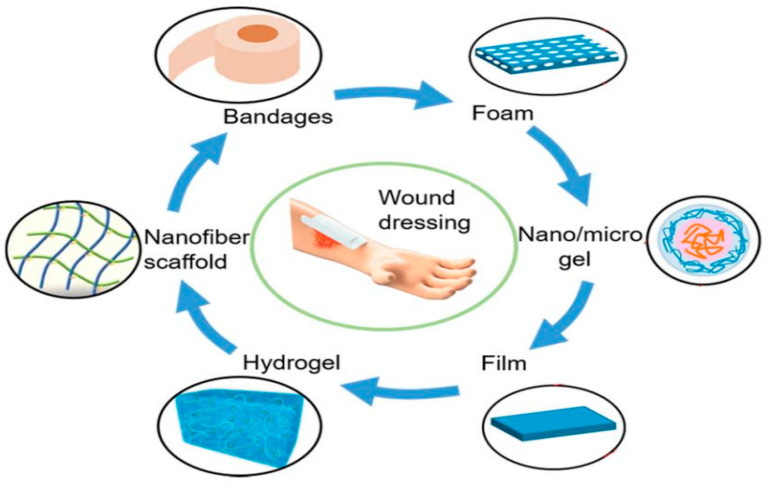
Different types of wound dressings. Reproduced with permission [98].

Wound dressings take a significant role in the process of wound healing, building a physical protective screen between the wound and the external environment, effectively preventing further hurt or infection from the outside world. Traditional dressings such as gauze have drawbacks such as difficulty in removal and the risk of secondary damage during use. Therefore, studying and preparing a new type of dressing based on bioactive materials is of great significance for wound treatment. As shown in the figure, most of the new wound dressings are applied to wound repair in the form of foam, film, hydrogel, and nanofiber scaffold (Figure 6) [97].

Traditional wound dressings have always had various issues such as its difficulty in promoting wound healing. At first, cellulose hydrogel only played the role of simple physical isolation and creating a humid state. With the increasing demand for wound repair, the requirements for material properties become higher and higher [98]. In the development of basic research, cellulose-based hydrogels, as a three-dimensional porous cross-linked polymer network, are widely used as wound dressings. First of all, the high water content not only makes the cellulose-based hydrogel wet the wound environment to reduce the risk of scar formation, but also brings a sense of coolness to people, thus reducing the pain of patients [99]. Cellulose-based hydrogel polymer networks in aqueous environments are often similar to the extracellular matrix (ECM) [100]. Therefore, it has the ability to repair wounds. Secondly, good swelling makes the hydrogel have excellent absorption ability, so that the hydrogel can absorb a large amount of tissue exudate [101]. Third, because the high porosity of the hydrogel promotes the transmission of oxygen, and promotes the gas exchange between the wound and the outside world, oxygen permeates and cools the wound, reduces the pain of the patient, and enables the tissue to “breathe” [102]. Fourth, hydrogels can show antibacterial properties through different methods, inhibit the reproduction of anaerobic bacteria in the wound, prevent the wound from being infected by bacteria [103], and provide a sterile environment for wound healing. In addition, the cellulose-based hydrogel with a porous structure can contain water in the serum, and it is easy to collect coagulation molecules, red blood cells, and platelets locally, so that the blood can coagulate faster [104]. 

**Figure 7:**
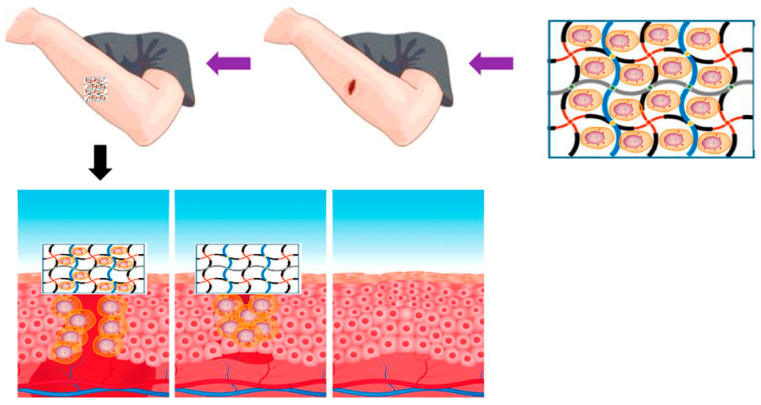
Typical wound healing of hydrogel based on nanocellulose in skin. Reproduced with permission [105].

Sajjad et al. [105] developed a nanocomposite material of BC (bacterial cellulose) and curcumin nanoparticles. BC/curcumin nanocomposites were used as wound dressing hydrogels, which effectively healed the burn wounds in the rat model. The antibacterial potential of curcumin was tested, and in vitro studies were conducted on Escherichia coli, Salmonella typhimurium, and Staphylococcus aureus using the pore diffusion method. The inhibitory regions were measured to determine the antibacterial potential of curcumin. The research results indicate that the combination of BC and curcumin can serve as an intelligent antibacterial wound dressing system to treat partial-thickness skin burns (Figure 7).

In conclusion, cellulose-based antibacterial hydrogel has a broad application prospect in wound dressings, which can provide multiple functions such as antibacterial and protection functions, and promoting healing, providing a new, efficient, and safe wound treatment method for the medical field.

### 6.2. Tissue Engineering

Cardiovascular disease has seriously threatened the health of modern people. Currently, introducing tissue-engineering treatment methods into the treatment of cardiovascular disease is considered one of the most promising options. Artificial synthetic materials such as polyester and polyurethane may cause inflammation or cause rejection reactions in the body. Therefore, it is necessary to find an effective matrix material suitable for tissue engineering. The preferred material for tissue engineering is natural polymer materials, which are non-toxic, and have good biocompatibility, biodegradability, and non-toxic side effects of the degradation products. Natural polymers also have a structure similar to or identical to the extracellular matrix environment, containing a large number of hydrophilic groups such as hydroxyl, amino, and carboxyl groups, which can promote cell adhesion, proliferation, and differentiation.

Cellulose is a polysaccharide with a crystalline structure, which is the most plentiful natural polymer in natural world. It has a high molecular weight, good mechanical properties, good water absorption performance, and good thermal stability. It has some unique advantages and potential in tissue engineering. One of the prime targets of tissue engineering is to exploit living, healthy, and functional tissues that can serve in tissue transplantation, or even in organ transplantation. Hydrogel is a special polymer, which can be swelled in water but not dissolved in water. This cross-linking network is made of many small molecular chains connected with each other. It has a high water content and elastic properties similar to tissue, making it an ideal scaffold for cell and tissue growth. Cells are loaded in a hydrogel network and implanted into the damaged parts of corresponding tissues or organs. Due to the excellent biocompatibility of hydrogels, they can be degraded and absorbed in the human body. Cells proliferate at the target location and secrete the extracellular matrix to develop into new tissues or organs, thus achieving the purpose of medical treatment.

There have been many relevant reports about the application of cellulose hydrogel in tissue engineering. Emilia Entcheva et al. tested the potential of cultivating and growing functional myocardial cells on cellulose acetate (CA) and regenerated cellulose (RC) scaffolds, and prepared functional cardiac-tissue-engineering scaffolds using cellulose as the matrix. They found that CA and RC scaffolds can promote the growth of myocardial cells, and enhance cell connectivity and electrophysiological function [106]. The combination of cellulose and other materials not only gives cellulose hydrogel new properties, but also broadens the application field of cellulose hydrogel. Jose et al. introduced a simple reverse template method to prepare hydrogels, demonstrated the applicability of this method in cellulose-based hydrogel scaffolds, and boldly speculated that this template strategy would be widely applicable to other soft water gel materials. The results indicate that it can promote its differentiation towards osteogenic outcomes, opening the door to the on-demand production of complex topological structures, and has broad application prospects in advanced and personalized biomimetic tissue engineering [107].

### 6.3. Bone Tissue

**Figure 8:**
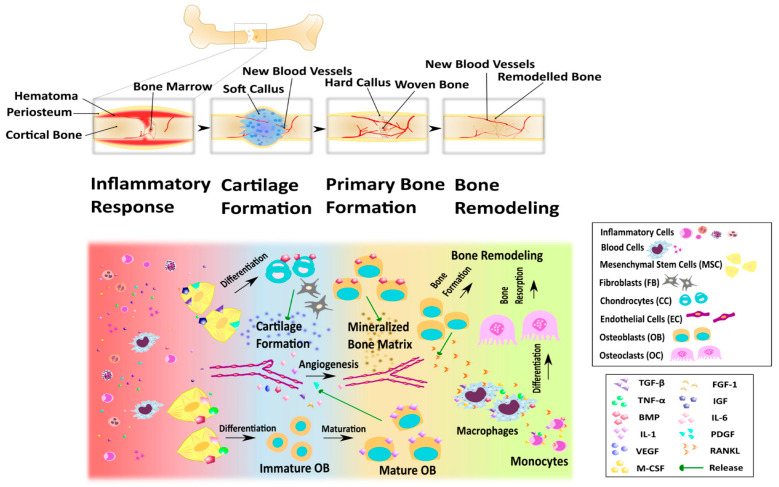
Stages of bone fracture healing. Upper part: changes that occur with the bone tissue during repair process; lower part: some of the cellular aspects and the factors that take part in the bone repair processes in each stage. Reproduced with permission.

Bone is a complex organ composed of specific cell types surrounded by the extracellular matrix, and its bioactive molecules are integrated or produced by cells. Natural fracture repair is a complex process that involves immune system activation, cell migration, differentiation, and apoptosis. Bone tissue can undergo natural regeneration over time. The process of bone tissue repair can be divided into four overlapping stages: inflammatory response, cartilage formation, primary bone formation, and bone reconstruction (Figure 8) [108]. Bone growth is a dynamic process, and bone defects that exceed the critical size threshold will not naturally heal. Severe bone injury can lead to large bone defects. In order to achieve functional recovery and complete healing, bone transplant substitutes are needed for reconstruction, promoting the process of bone healing. Many treatment methods, with limited availability and lack of materials, can have side effects including immune rejection and the spread of infectious pathogens. Compared with common tissue repair materials such as biological scaffolds, microspheres, and films, hydrogels have the advantages of having a soft, wet surface and low friction coefficient as bone-tissue-engineering materials. In terms of biological properties, hydrogels can provide a friendly environment for the active factors and cells needed for bone tissue repair [109]; the interaction between different biomaterials makes the hydrogel have a certain mechanical strength. Hydrogels used for bone tissue engineering need to have mechanical properties and a structure similar to bone tissue, have an affinity with the bone tissue contact surface, and can load a large number of bone tissue active cells and growth factors and other microbial materials to promote bone regeneration, which can provide an ideal growth environment for microbial carriers. Hydrogels have been used as implants for cartilage and bone repair [110]. In the field of fracture or bone defect repair, natural polymers and their derivatives can be used to prepare hydrogels. Research shows that hydrogels formed by these natural polymer materials have good biocompatibility, cytotoxicity, and plasticity, and encourage the adhesion and proliferation of bone cells, providing a good growth environment for bone regeneration.

**Figure 9:**
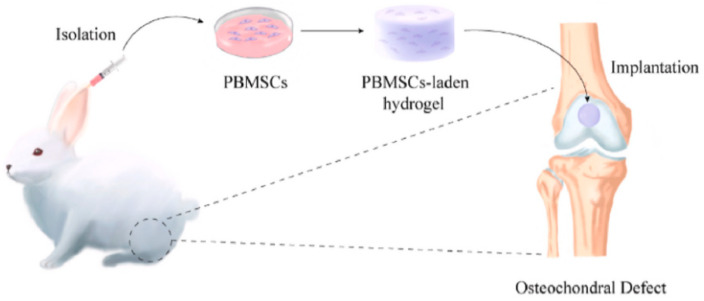
Operation flow chart of separation, encapsulation, and hydrogel implantation into osteochondral defects. Reproduced with permission [111].

Yang et al. [111]. prepared a new type of polylysine hydrogel through in situ cross-linking by using thiol/thioester functionalized hyperbranched polyamino acids. After implanting peripheral blood MSCs loaded with it into a rabbit cartilage defect model, it showed excellent repair effects. Micro CT showed that the effect of subchondral bone repair in the hydrogel group was better than that in the blank control group. Further research found that the cells infiltrating around the polylysine hydrogel were mainly M2 macrophages, which showed the importance of immune regulation in cartilage tissue repair. The polylysine hydrogel carrying stem cells had potential application prospects in the field of bone and cartilage regeneration (Figure 9).

### 6.4. Self-Healing

The emergence of intelligent materials not only effectively liberates labor productivity, but also brings great convenience and comfort to human life. Self-repairing cellulose-based hydrogel is one of the smart materials, and the study of its antibacterial properties has become a hot spot in the current high-tech field. However, the preparation of traditional self-healing hydrogels mostly requires the use of fossil resources to synthesize organic polymers, which does not conform to the concept of green and sustainable development. Moreover, due to the potential toxicity, its application in biological, medical, and other fields will be limited. Using natural polymer (cellulose) instead of synthetic polymer to prepare the self-repairing hydrogel is expected to completely solve the above problems. The main reason is that the good affinity of cellulosic materials to cells/tissues can effectively improve the biocompatibility of self-repairing hydrogels, thus making them suitable for biological, medical, and other fields; in addition, the rich functional groups in the cellulose macromolecular chain and the rich modification methods involved make the processability of cellulose have obvious advantages over other biomass, which is more suitable for the preparation of self-healing composite hydrogels.

First, cellulose-based self-healing hydrogel can interact with the extracellular matrix to provide a suitable growth environment for cells and promote cell adhesion and proliferation. Due to its self-healing properties, it can self-repair and regenerate at the site of injury, providing an effective therapeutic approach for tissue engineering. Secondly, cellulose-based self-healing hydrogels can be used as drug carriers to wrap drugs in them, and slow drug release can be achieved by controlling the dissolution rate of hydrogels. This hydrogel can effectively kill the pathogenic bacteria, reduce the risk of infection, and provide a guarantee for the healing process of tissue engineering. Meanwhile, due to its self-healing performance, it can automatically repair the damaged area during drug release, keeping the wound moist and clean, and providing a better environment for wound healing. In addition, cellulose-based self-healing hydrogels can also promote tissue repair and regeneration by regulating their physical and chemical properties, such as water absorption, permeability, flexibility, and loading bioactive ingredients. For example, it can be used to prepare tissue-engineering products such as artificial skin and joints, providing patients with better treatment options. Meanwhile, due to its self-healing performance, it can automatically adapt to body shape and exercise status during use, providing better protection for high-intensity sports groups such as athletes. In a word, cellulose-based self-healing hydrogel, as a new type of biomaterial, has a broad application prospect and contributes to the improvement of human health and quality of life.

The development of the cellulose self-healing composite hydrogel can not only realize the high value-added utilization of biomass resources, but also ensure resource security, reduce environmental pollution, and realize the harmonious coexistence of humans and nature. In recent years, although self-healing hydrogels based on different biomass have been developed, problems such as the low mechanical properties or long self-healing recovery period of gel fracture generally exist, which fail to give full play to its advantages. The preparation of self-healing/shape memory hydrogels from nanocellulose is expected to improve this situation. The excellent mechanical strength of nanocellulose can endow hydrogel with a significant enhancement effect: a higher specific surface area, abundant functional groups, and modification methods can ensure the efficient introduction of multiple self-healing mechanisms; in addition, the excellent dispersion stability of cellulose can be used as a dispersant of multifunctional nanoparticles to realize the multifunctional hydrogel. Therefore, the preparation of the self-repairing hydrogel with nanocellulose as the raw material can not only effectively improve the mechanical stability, processability, and biocompatibility of hydrogel, but also realize the high value-added utilization of biomass resources, ensure resource security, and reduce environmental pollution.

### 6.5. Skin

As the largest organ in the human body, composed of the epidermis, dermis, subcutaneous tissue, and its accessory organs (sweat glands, sebaceous glands, blood vessels, etc.), with various functions such as as a physical barrier, immune defense, and temperature regulation [112]. Its main function is to act as an external barrier, protecting internal organs from the external environment [113]. But it is also highly susceptible to external factors, and many systemic diseases can cause skin damage, which may form a complex wound environment, making it easier to cause wound infection and induce excessive inflammatory reactions due to the accumulation of inflammatory factors. These factors may pose a threat to the repair cells around the wound, forming chronic wounds and hindering wound healing. Skin tissue diseases are a global public health issue, and chronic wounds will continue to be an increasingly persistent problem among this population. In particular, chronic unhealed wounds are particularly susceptible to coronavirus disease (COVID-19) [114]. Therefore, reasonable wound treatment plays a crucial role in repairing damaged tissues and improving the quality of life of patients with wound damage. At present, the clinical treatment method for skin injuries is skin transplantation. Due to the limited area of skin tissue that can be transplanted in the human body, it is limited by hygiene, safety, and other aspects. Therefore, traditional repair methods are very limited in the treatment of large-scale skin injuries. The development of tissue-engineering technology provides new technological solutions for skin injury repair. Skin tissue engineering utilizes biodegradable scaffolds that can be degraded and absorbed by the human body to carry cells. In different tissue-engineering scaffolds, cellulose-based hydrogels are particularly interesting because their structures are similar to extracellular matrix, which can provide a humid environment and porous structure. In addition, hydrogel has the ability of hydration healing, and has appropriate oxygen permeability and antibacterial properties. At the same time, it absorbs wound exudates, prevents bacterial infection, improves epithelial formation, and provides an environment for tissue regeneration [115]. Cellulose and its derivatives have excellent mechanical properties and high water absorption, and contain a large number of hydroxyl groups, which is conducive to forming composite hydrogels with other polymers or small molecules, and are good scaffold materials for tissue engineering.

**Figure 10:**
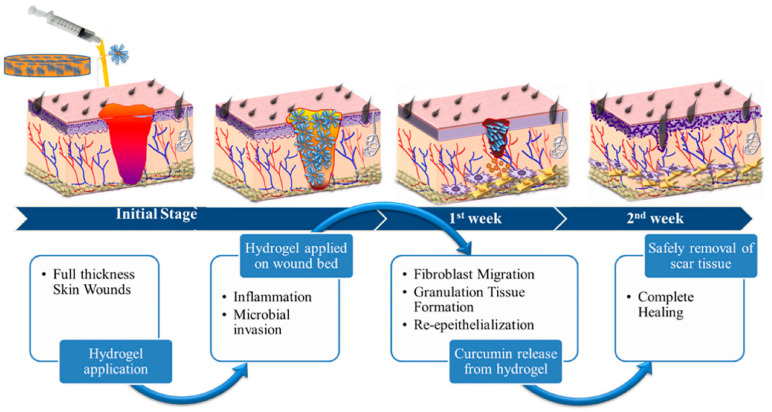
Wound-healing mechanism at the same stage after application of hydrogel. Reproduced with permission [116].

Shefa et al [116] prepared physically hydrogen-bonded cross-linked TOCN polyvinyl alcohol (PVA) curcumin (Cur) by the freeze thaw method. The hydrogel can release Cur with antibacterial, anti-inflammatory, and antioxidant functions to promote wound healing. It can be seen that the wound-healing mechanism of hydrogel in Figure 10 occurs at different stages. After two weeks of hydrogel treatment, there was the obvious formation of new epidermis and granulation tissue in the defect area, and collagen fibers gathered near the defect area, indicating that TOCN-PVA-Cur hydrogel can effectively promote wound healing.

Huang et al. [117] used lignocellulose as the raw material to prepare CNC through sulfuric acid hydrolysis, and then prepared dialdehyde-modified CNC through periodic acid oxidation. A novel nanocomposite hydrogel was constructed by the combining flexible carboxymethyl chitosan (CMC) chain and rigid carboxymethyl chitosan (DAC NC) chain through dynamic cross-linking. The gel has high self-healing efficiency, high injectivity, good mechanical strength, and a balanced swelling rate. In addition, amino acid will cause the decomposition of the hydrogel, so it can be dissolved in amino acid solution as required, so that they can be removed painlessly when changing wound dressings. In vivo experiments showed that the injectable self-healing hydrogel was effective in treating deep second-degree burn wounds without scar formation. L929 cells (mouse fibroblasts) were cultured with hydrogel G/THPZB/Fe designed by Li et al. [118]. The membrane skeleton and morphology were long and spindle-shaped under confocal microscope, which proved that it had cell adhesion, thus promoting the proliferation of L929 cells. Hydrogels can also improve the microenvironment of wounds and regulate the secretion of cell differentiation and growth-related factors.

## 7. Conclusions

Over the past few decades, remarkable progress has been made in the field of biomaterials, especially in the development of novel antimicrobial materials. Cellulose-based antibacterial hydrogels, as a new biomaterial, have been garnered widespread interest for their unique advantages and potential. Cellulose is a kind of natural polymer material with extensive sources and abundant reserves. It is non-toxic and harmless to the human body, does not cause an immune reaction, and has the advantages of having a low cost, stable mechanical properties, good water absorption, and water retention, making cellulose the most suitable for the antibacterial role of hydrogel carrier. Firstly, the preparation method of cellulose-based hydrogel was discussed. Two main methods of chemical and physical cross-linking are introduced. The chemical modification of cellulose is mainly through esterification, free radical polymerization, addition reaction, etc. Physical cross-linking mainly uses the unique properties of natural cellulose, such as hydrogen bonding and hydrophobic interactions, to form hydrogels with antibacterial properties. Secondly, the application and characteristics of several typical cellulose-based antibacterial hydrogels were summarized, including supported metal nanoparticles, metal oxide nanoparticles, and so on. The application of cellulose-based antibacterial hydrogels in wound dressing, tissue engineering, and bone tissue was reviewed in detail. However, with the continuous deepening and development of research technology, and the emergence of bacterial resistance due to the overuse of antibiotics, cellulose-based antibacterial hydrogels must have new biomedical functions such as a higher antibacterial ability, promoting tissue regeneration and wound healing to solve these problems. Despite the significant advantages of cellulose-based antibacterial hydrogels in terms of antibacterial properties, their practical application still faces some challenges, for example, how to further improve its antibacterial performance, how to achieve large-scale production and application, how to ensure long-term stability and safety. With the increasing demand for biological materials, we believe that cellulose-based antibacterial hydrogels will play an increasingly important role in medical, environmental protection, food, and other fields. For example, it can be used to prepare antibacterial dressings, biosensors, environmental purification materials, etc. At the same time, we also expect more research to focus on improving its antibacterial performance, achieving large-scale production and application, and ensuring long-term stability and safety. Therefore, we believe that cellulose-based antibacterial hydrogels will be more widely used, and more in-depth research should be conducted in the future.

## Data Availability

Not applicable.

## References

[1] Srivastava P., Kim K.-S. (2022). Membrane Vesicles Derived from Gut Microbiota and Probiotics: Cutting-Edge Therapeutic Approaches for Multidrug-Resistant Superbugs Linked to Neurological Anomalies. Pharmaceutics.

[2] Blair J.M.A., Webber M.A., Baylay A.J., Ogbolu D.O., Piddock L.J.V. (2015). Molecular mechanisms of antibiotic resistance. Nat. Rev. Microbiol..

[3] Sutherland E.R., Martin R.J. (2007). Asthma and Atypical Bacterial Infection. Chest.

[4] Li S., Dong S., Xu W., Tu S., Yan L., Zhao C., Ding J., Chen X. (2018). Antibacterial Hydrogels. Adv. Sci..

[5] Salick D.A., Kretsinger J.K., Pochan D.J., Scheider J.P. (2007). Inherent Antibacterial Activity of a Peptide-Based β-Hairpin Hydrogel. J. Am. Chem. Soc..

[6] Wang K., Hao Y.T., Wang Y., Chen J., Mao L., Deng Y., Chen J.I., Yuan S., Zhang T., Ren J. (2019). Functional Hydrogels and Their Application in Drug Delivery, Biosensors, and Tissue Engineering. Int. J. Polym. Sci..

[7] Hook A.L., Chang C.-Y., Yang J., Atkinson S., Langer R., Anderson D.G., Davies M.C., Williams P., Alexander M.R. (2013). Discovery of Novel Materials with Broad Resistance to Bacterial Attachment Using Combinatorial Polymer Microarrays. Adv. Mater..

[8] Ahamed M., Alsalhi M.S., Siddiqui M.K.J. (2010). Silver Nanoparticle Applications and Human Health. Clini. Chim. Acta.

[9] Rosenboom J.G., Langer R., Traverso G. (2022). Bioplastics for a circular economy. Nat. Rev. Mater..

[10] Peng Z., Lin Q., Tai Y.A., Wang Y. (2020). Applications of Cellulose Nanomaterials in Stimuli-Responsive Optics. Agric. Food. Chem..

[11] Loelovich M. (2021). Adjustment of Hydrophobic Properties of Cellulose Materials. Polymers.

[12] Srivastava D., Kuklin M.S., Ahopelto J., Karttunen A.J. (2020). Electronic band structures of pristine and chemically modified cellulose allomorphs. Carbohyd. Polym..

[13] Mehran A. (2019). Modifications of microcrystalline cellulose (MCC), nanofibrillated cellulose (NFC), and nanocrystalline cellulose (NCC) for antimicrobial and wound healing applications. E-Polymers.

[14] Jarvis M.C. (2018). Structure of native cellulose microfibrils, the starting point for nanocellulose manufacture. Phil. Trans. R. Soc..

[15] Popescu M.-C., Dogaru B.-I., Popescu C.-M. (2020). Effect of Cellulose Nanocrystals Nanofiller on the Structure and Sorption Properties of Carboxymethyl Cellulose–Glycerol–Cellulose Nanocrystals Nanocomposite Systems. Materials.

[16] Ioelovich M., Leykin A. (2011). Study of sorption properties of cellulose and its derivatives. Bioresources.

[17] Yuk H., Lu B., Zhao X. (2019). Hydrogel bioelectronics. Chem. Soc. Rev..

[18] Chen G., Tang W., Wang X., Zhao X., Chen C., Zhu Z. (2019). Applications of Hydrogels with Special Physical Properties in Biomedicine. Polymers.

[19] Zhang Y.F., Yu T.T., Peng L.Y., Sun O.N., Wei Y., Han B. (2020). Advancements in Hydrogel-Based Drug Sustained Release Systems for Bone Tissue Engineering. Front. Pharmacol..

[20] Yang K.R., Han Q., Chen B., Zheng Y., Zhang K., Li Q., Wang J.C. (2018). Antimicrobial hydrogels: Promising materials for medical application. Int. J. Nanomed..

[21] Liu J., Jiang W., Xu Q., Zheng Y. (2022). Progress in Antibacterial Hydrogel Dressing. Gels.

[22] Li Y.S., Han Y., Qin J.T., Song Z.Y., Cai H.H., Du J.F., Sun S.F., Liu Y. (2016). Photosensitive antibacterial and cytotoxicity performances of a TiO_2_/carboxymethyl chitosan/poly(vinyl alcohol) nanocomplex hydrogel byin situradiation construction. J. Appl. Polym. Sci..

[23] Zhang H., Zhu J., Hu Y., Chen A., Zhou L., Gao H., Liu Y., Liu S. (2019). Study on photocatalytic antibacterial and sustained-release properties of cellulose/TiO_2_/β-CD complex hydrogel. J. Nanomater..

[24] Qu J., Li J., Zhu W., Xu Y., Zhou S., Yang Y., Qian X. (2022). Hybrid nanocomposite multinetwork hydrogel containing magnesium hydroxide nanoparticles with enhanced antibacterial activity for wound dressing applications. Polymer.

[25] Yang Z., Xu C.P., Chen Y., Li W., Wang L., Yuan Z.G. (2021). A novel mechanical-based injective hydrogel for treatment with aromatase inhibitors caused joint inflammation via the NF-kappaB pathway. ACS Omega.

[26] Suljovrujic E., Miladinovic Z.R., Micic M., Suljovrujic D., Milicevic D. (2019). The influence of monomer/solvent feed ratio on POEGDMA thermoresponsive hydrogels: Radiation-induced synthesis, swelling properties and VPTT. Radiat. Phys. Chem..

[27] Patwa R., Zandraa O., Capáková Z., Saha N., Sáha P. (2020). Effect of Iron-Oxide Nanoparticles Impregnated Bacterial Cellulose on Overall Properties of Alginate/Casein Hydrogels: Potential Injectable Biomaterial for Wound Healing Applications. Polymers.

[28] Luthfianti H.R., Waresindo W.X., Edikresnha D., Chahyadi A., Suciati T., Noor F.A., Khairurrijal K. (2023). Physicochemical characteristics and antibacterial activities of freeze-thawed polyvinyl alcohol/andrographolide hydrogels. ACS Omega.

[29] Huo J., Jia Q., Huang H., Zhang J., Li P., Dong X., Huang W. (2021). Emerging photothermal-derived multimodal synergistic therapy in combating bacterial infections. Chem. Soc. Rev..

[30] Fan X., Zhao L., Ling Q., Liu J., Gu H. (2022). Mussel-induced nano-silver antibacterial, self-healing, self-adhesive, anti-freezing, and moisturizing dual-network organohydrogel based on SA-PBA/PVA/CNTs as flexible wearable strain sensors. Polymer.

[31] Dong Z., Lin Y., Xu S., Linna C., Zhao X., Mei X., Gao X. (2023). NIR-triggered tea polyphenol-modified gold nanoparticles-loaded hydrogel treats periodontitis by inhibiting bacteria and inducing bone regeneration. Mater. Des..

[32] Wang Y., Yao H., Zu Y., Yin W. (2022). Biodegradable MoOx@MB incorporated hydrogel as light-activated dressing for rapid and safe bacteria eradication and wound healing. RSC Adv..

[33] Huang B., Hu D., Dong A., Tian J., Zhang W. (2022). Highly antibacterial and adhesive hyaluronic acid hydrogel for wound repair. Biomacromolecules.

[34] Farnaz A., Ali Reza K. (2022). Injectable photosensitizing supramolecular hydrogels: A robust physically cross-linked system based on polyvinyl alcohol/chitosan/tannic acid with self-healing and antioxidant properties. React. Funct. Polym..

[35] Laurano R., Boffito M. (2020). Thermosensitive micellar hydrogels as vehicles to deliver drugs with different wettability. Front. Bioeng. Biotechnol..

[36] Li W. (2020). Supramolecular nanofiber-reinforced Puerarin hydrogels as drug carriers with synergistic controlled release and antibacterial properties. J. Mater. Sci..

[37] Zhang Q., Ren T., Gan J., Sun L., Guan C., Zhang Q., Pan S., Chen H. (2022). Synthesis and Rheological Characterization of a Novel Salecan Hydrogel. Pharmaceutics.

[38] Fatema N., Ceballos R.M., Fan C. (2022). Modifications of cellulose-based biomaterials for biomedical applications. Front. Bioeng. Biotechnol..

[39] Korhonen O., Budtova T. (2019). Gelation of cellulose-NaOH solutions in the presence of cellulose fibers. Carbohydr. Polym..

[40] Clarkson C.M., El Awad S.M., Forti E.S., Schueneman G.T., Moon R.J., Youngblood J.P. (2021). Recent Developments in Cellulose Nanomaterial Composites. Adv. Mater..

[41] Shen X.P., Shamshina J.L., Berton P., Gurau G., Rogers R.D. (2016). Hydrogels based on cellulose and chitin: Fabrication, properties, and applications. Green. Chem..

[42] Xu X., Hu Y., Wang S.C., Chen X., Jiang Y.Y., Su J.C. (2022). Fabrication of physical and chemical crosslinked hydrogels for bone tissue engineering. Bio. Mater..

[43] Wang Q.H., Zhang Y., Ma Y., Pan G.Q. (2023). Nano-crosslinked dynamic hydrogels for biomedical applications. Mater. Today Bio..

[44] Liu X., Pang J., Zhang X., Wu Y., Sun R. (2013). Regenerated cellulose film with enhanced tensile strength prepared with ionic liquid 1-ethyl-3-methylimidazolium acetate (EMIMAc). Cellulose.

[45] Shao C., Yang J. (2020). Dynamics in cellulose-based hydrogels with reversible cross-links. Self-Heal. Self-Recover. Hydrogels.

[46] Jung H.S., Kim H.C., Prak W.H. (2019). Robust methylcellulose hydrogels reinforced with chitin nanocrystals. Carbohyd. Polym..

[47] Kaiser P., Werner M., Jérôme V., Hübner H., Buchholz R., Freitag R. (2014). Cell retention by encapsulation for the cultivation of Jurkat cells in fixed and fluidized bed reactors. Biotechnol. Bioeng..

[48] Müller M., Keßler B. (2012). Release of pamidronate from poly(ethyleneimine)/cellulose sulphate complex nanoparticle films: An in situ ATR-FTIR study. Pharm. Biomed. Anal..

[49] Lu A., Wang Y., Boluk Y. (2014). Investigation of the scaling law on gelation of oppositely charged nanocrystalline cellulose and polyelectrolyte. Carbohydr. Polym..

[50] Hussain I., Sayed S.M., Liu S., Oderinde O., Kang M., Yao F., Fu G. (2018). Enhancing the mechanical properties and self-healing efficiency of hydroxyethyl cellulose-based conductive hydrogels via supramolecular interactions. Eur. Polym. J..

[51] Hennink W.E., Van Nostrum C.F. (2012). Novel crosslinking methods to design hydrogels. Adv. Drug Deliv. Rev..

[52] Duquette D., Dumont M.J. (2019). Comparative studies of chemical crosslinking reactions and applications of bio-based hydrogels. Polym. Bull..

[53] Maity J., Ray S.K. (2017). Removal of Cu (II) ion from water using sugar cane bagasse cellulose and gelatin based composite hydrogels. Int. J. Biol. Macromol..

[54] Rao K.M., Kumar A., Han S.S. (2017). Poly(acrylamidoglycolic acid)nanocomposite hydrogels reinforced with cellulose nanocrystals for pH-sensitive controlled release of diclofenac sodium. Polym. Test..

[55] Kono H., Fujita S. (2012). Biodegradable superabsorbent hydrogels derived from cellulose by esterification crosslinking with 1,2,3,4-butanetetracarboxylic dianhydride. Carbohydr. Polym..

[56] Demitri C., Del Sole R., Scalera F., Sannino A., Vasapollo G., Maffezzoli A., Ambrosio L., Nicolais L. (2008). Novel superabsorbent cellulose-based hydrogels crosslinked with citric acid. Appl. Polym. Sci..

[57] Senna A.M., Novack K.M., Botaro V.R. (2014). Synthesis and characterization of hydrogels from cellulose acetate by esterification crosslinking with EDTA dianhydride. Carbohydr. Polym..

[58] Esposito A., Sannino A., Cozzolino A., Quintiliano S.N., Lamberti M., Ambrosio L., Nicolais L. (2005). Response of intestinal cells and macrophages to an orally administered cellulose-PEG based polymer as a potential treatment for intractable edemas. Biomaterials.

[59] Butun S., Ince F.G., Erdugan H., Sahiner N. (2011). One-step fabrication of biocompatible carboxymethyl cellulose polymeric particles for drug delivery systems. Carbohydr. Polym..

[60] Tavakolian M., Munguia-Lopez J.G., Valiei A., Islam M.S., Kinsella J.M., Tufenkji N., Van de Ven T.G.M. (2020). Highly absorbent antibacterial and biofilm-disrupting hydrogels from cellulose for wound dressing applications. ACS Appl. Mater. Interfaces.

[61] Oyarzun-Ampuero F., Vidal A., Concha M., Morales J., Orellana S., Moreno-Villoslada I. (2015). Nanoparticles for the treatment of wounds. Curr. Pharm. Des..

[62] Liang D., Lu Z., Yang H., Gao J., Chen R. (2016). Novel asymmetric wettable AgNPs/Chitosan Wound Dressing: In Vitro and In Vivo Evaluation. ACS Appl. Mater. Interfaces.

[63] Mokhena T.C., Luyt A.S. (2017). Electrospun alginate nanofibres impregnated with silver nanoparticles: Preparation, morphology and antibacterial properties. Carbohydr. Polym..

[64] Shao W., Wu J., Wang S., Huang M., Liu X., Zhang R. (2017). Construction of silver sulfadiazine loaded chitosan composite sponges as potential wound dressings. Carbohydr. Polym..

[65] Bao Y., He J., Song K., Guo J., Zhou X., Liu S. (2021). Plant-extract-mediated synthesis of metal nanoparticles. J. Chem..

[66] Celebioglu A., Aytac Z., Umu O.C.O., Dana A., Tekinay T., Uyar T. (2014). One-step synthesis of size-tunable Ag nanoparticles incorporated in electrospun PVA/cyclodextrin nanofibers. Carbohydr. Polym..

[67] Csoka L., Bozanic D.K., Nagy V., Dimitrijevic-Brankovic S., Luyt A.S., Grozdits G., Djokovic V. (2012). Viscoelastic properties and antimicrobial activity of cellulose fiber sheets impregnated with Ag nanoparticles. Carbohydr. Polym..

[68] Emam H.E., Mowafi S., Mashaly H.M., Rehan M. (2014). Production of antibacterial colored viscose fibers using in situ prepared spherical Ag nanoparticles. Carbohydr. Polym..

[69] Lavorgna M., Attianese I., Buonocore G.G., Conte A., Del Nobile M.A., Tescione F., Amendola E. (2014). MMT-supported Ag nanoparticles for chitosan nanocomposites: Structural properties and antibacterial activity. Carbohydr. Polym..

[70] Qu F., Ding Y., Lv X., Xia L., You J., Han W. (2019). Emissions of terbium metal-organic frameworks modulated by dispersive/agglomerated gold nanoparticles for the construction of prostate-specific antigen biosensor. Anal. Bioanal. Chem..

[71] Al-Enizi A.M., Ahamad T., Al-Hajji A.B., Ahmed J., Chaudhary A.A., Alshehri S.M. (2018). Cellulose gum and copper nanoparticles based hydrogel as antimicrobial agents against urinary tract infection (UTI) pathogens. Int. Biol. Macromol..

[72] Bundjaja V., Santoso S.P., Angkawijaya A.E., Yuliana M., Soetaredjo F.E., Ismadji S., Ayucitra A., Gunarto C., Ju Y.-H., Ho M.-H. (2021). Fabrication of cellulose carbamate hydrogel-dressing with rarasaponin surfactant for enhancing adsorption of silver nanoparticles and antibacterial activity. Mater. Sci. Eng. C-Mater. Biol. Appl..

[73] Bao Y., He J., Song K., Guo J., Zhou X., Liu S. (2022). Functionalization and Antibacterial Applications of Cellulose-Based Composite Hydrogels. Polymers.

[74] Jones N., Ray B., Ranjit K.T., Manna A.C. (2008). Antibacterial activity of ZnO nanoparticle suspensions on a broad spectrum of microorganisms. FEMS Microbiol. Lett..

[75] James C., Pugh T., Johnson A.L., Jenkins A.T.A. (2011). An antimicrobial zinc based molecule for cross linking polyacrylic acid. Eur. Polym. J..

[76] Sabbagh F., Muhamad I.I. (2017). Acrylamide-based hydrogel drug delivery systems: Release of acyclovir from MgO nanocomposite hydrogel. J. Taiwan Inst. Chem. Eng..

[77] Dizaj S.M., Lotfipour F., Barzegar-Jalali M., Zarrintan M.H., Adibkia K. (2014). Antimicrobial activity of the metals and metal oxide nanoparticles. Mater. Sci. Eng. C-Mater. Biol. Appl..

[78] Hrenovic J., Milenkovic J., Daneu N., Kepcija R.M., Rajic N. (2012). Antimicrobial activity of metal oxide nanoparticles supported onto natural clinoptilolite. Chemosphere.

[79] Song J., Yuan C., Jiao T., Xing R., Yang M., Adams D., Yan X. (2020). Multifunctional Antimicrobial Biometallohydrogels Based on Amino Acid coordinated self-Aassembly. Small.

[80] Yadollahi M., Gholamali I., Namazi H., Aghazadeh M. (2015). Synthesis and characterization of antibacterial carboxymethyl cellulose/ZnO nanocomposite hydrogels. Int. J. Biol. Macromol..

[81] George D., Maheswari P.U., Sheriffa Begum K.M.M.S., Arthanareeswaran G. (2019). Biomass-derived dialdehyde cellulose cross-linked chitosan-based nanocomposite hydrogel with phytosynthesized zinc oxide nanoparticles for enhanced curcumin delivery and bioactivity. J. Agric. Food Chem..

[82] Gao W., Chen Y., Zhang Y., Zhang Q., Zhang L. (2018). Nanoparticle-based local antimicrobial drug delivery. Adv. Drug Deliv. Rev..

[83] Hoque J., Bhattacharjee B., Prakash R.G., Paramanandham K., Haldar J. (2018). Dual function injectable hydrogel for controlled release of antibiotic and local antibacterial therapy. Biomacromolecules.

[84] Taccone F.S., Bond O., Cavicchi F.Z., Hites M. (2016). Individualized antibiotic strategies. Curr. Opin. Anesth..

[85] Sattari S., Tehrani A.D., Adeli M. (2018). PH-responsive hybrid hydrogels as antibacterial and drug delivery systems. Polymers.

[86] Xu W., Dong S., Han Y., Li S., Liu Y. (2018). Hydrogels as antibacterial biomaterials. Curr. Pharm. Des..

[87] Forero-Doria O., Polo E., Marican A., Guzman L., Venegas B., Vijayakumar S., Wehinger S., Guerrero M., Gallego J., Duran-Lara E.F. (2020). Supramolecular hydrogels based on cellulose for sustained release of therapeutic substances with antimicrobial and wound healing properties. Carbohydr. Polym..

[88] Iman M., Barati A., Safari S. (2019). Characterization, in vitro antibacterial activity, and toxicity for rat of tetracycline in a nanocomposite hydrogel based on PEG and cellulose. Cellulose.

[89] Antunes J.C., Domingues J.M., Miranda C.S., Silva A.F.G., Homem N.C., Amorim M.T.P., Felgueiras H.P. (2021). Bioactivity of chitosan-based particles loaded with plant-derived extracts for biomedical applications: Emphasis on antimicrobial fiber-based systems. Mar. Drugs.

[90] Friedman M. (2015). Antibiotic-resistant bacteria: Prevalence in food and inactivation by food-compatible compounds and plant extracts. Agric. Food Chem..

[91] Ge W., Cao S., Shen F., Wang Y., Ren J., Wang X. (2019). Rapid self-healing, stretchable, moldable, antioxidant and antibacterial tannic acid-cellulose nanofibril composite hydrogels. Carbohydr. Polym..

[92] Ravindra S., Mulaba Bafubiandi A.F., Rajinikanth V., Varaprasad K., Reddy N.N., Raju K.M. (2012). Development and characterization of curcumin loaded silver nanoparticle hydrogels for antibacterial and drug delivery applications. J. Inorg. Organomet. Polym. Mater..

[93] Oryan A., Alemzadeh E., Moshiri A. (2016). Biological properties and therapeutic activities of honey in woundhealing: A narrative review and meta-analysis. J. Tissue Viability.

[94] Nho Y.C., Park J.S., Lim Y.M. (2014). Preparation of hydrogel by radiation for the healing of diabetic ulcer. Radiat. Phys. Chem..

[95] Omali N.B., Subbaraman L.N., Coles Brennan C., Fadli Z., Jones L.W. (2015). Biological and clinical implications of lysozyme deposition on soft contact lenses. Optom. Vision Sci..

[96] Wang X., Qiu L., Wang C., Gao Z.H., Zhou S.W., Cui P.F., Jiang P.J., Hu H.Z., Ni X.Y., Du X.C. (2022). Nanodot-doped peptide hydrogels for antibacterial phototherapy and wound healing. Biomater. Sci..

[97] Liu X., Xu H., Zhang M., Yu D.-G. (2021). Electrospun Medicated Nanofibers for Wound Healing: Review. Membranes.

[98] Hu H., Xu F.J. (2020). Rational design and latest advances of polysaccharide-based hydrogels forwound healing. Biomater. Sci..

[99] Kumar A., Kaur H. (2020). Sprayed in-situ synthesis of polyvinyl alcohol/chitosan loaded silver nanocomposite hydrogel for improved antibacterial effects. Int. J. Biol. Macromol..

[100] Sun X., Ma C., Gong W., Ma Y., Ding Y., Liu L. (2020). Biological properties of sulfanilamide-loaded alginate hydrogel fibers based on ionic and chemical crosslinking for wound dressings. Int. J. Biol. Macromol..

[101] Singh B., Kumar A. (2020). Graft and crosslinked polymerization of polysaccharide gum to form hydrogel wound dressings for drug delivery applications. Carbohyd. Res..

[102] Kalantari K., Mostafavi E., Saleh B., Soltantabar P., Webster T.J. (2020). Chitosan/pva hydrogels incorporated with green synthesized cerium oxide nanoparticles for wound healing applications. Eur. Polym. J..

[103] De Cicco F., Reverchon E., Adami R., Auriemma G., Russo P., Calabrese E.C., Porta A., Aquino R.P., Del Gaudio P. (2014). In situ forming antibacterial dextran blend hydrogel for wound dressing: SAA technology vs. spray drying. Carbohyd. Polym..

[104] Li S., Chen N., Li X., Li Y., Xie Z., Ma Z., Zhao J., Hou X., Yuan X. (2020). Bioinspired double-dynamic-bond crosslinked bioadhesive enables post-wound closure care. Adv. Funct. Mater..

[105] Sajjad W., He F., Ullah M.W., Ikram M., Shah S.M., Khan R., Khan T., Khalid A., Yang G., Wahid F. (2020). Fabrication of Bacterial Cellulose-Curcumin Nanocomposite as a Novel Dressing for Partial Thickness Skin Burn. Front. Bioeng. Biotechnol..

[106] Entcheva E., Bien H., Yin L., Chung C., Farrell M., Kostov Y. (2004). Functional cardiac cell constructs on cellulose-based scaffolding. Biomaterials.

[107] Torres-Rendon J.G., Femmer T., De Laporte L., Tigges T., Rahimi K., Gremse F., Zafarnia S., Lederle W., Ifuku S., Wessling M. (2015). Bioactive Gyroid Scaffolds Formed by Sacrificial Templating of Nanocellulose and Nanochitin Hydrogels as Instructive Platforms for Biomimetic Tissue Engineering. Adv. Mater..

[108] Witzler M., Büchner D., Shoushrah S.H., Babczyk P., Baranova J., Witzleben S., Tobiasch E., Schulze M. (2019). Polysaccharide-Based Systems for Targeted Stem Cell Differentiation and Bone Regeneration. Biomolecules.

[109] Stevens M., George J. (2005). Exploring and Engineering the Cell Surface Interface. Science.

[110] Xue X., Hu Y., Deng Y., Su J.C. (2021). Recent Advances in Design of Functional Biocompatible Hydrogels for Bone Tissue Engineering. Adv. Funct. Mater..

[111] Yang M., Zhang Z.C., Yuan F.Z., Deng R.H., Yan X., Mao F.B., Chen Y.R., Lu H., Yu J.K. (2022). An immunomodulatory polypeptide hydrogel for osteochondral defect repair. Bioact. Mater..

[112] Azimi B., Maleki H., Zavagna L., De la Ossa J.G., Linari S., Lazzeri A., Danti S. (2020). Bio-Based Electrospun Fibers for Wound Healing. J. Funct. Biomater..

[113] Bacakova L., Pajorova J., Bacakova M., Skogberg A., Kallio P., Kolarova K., Svorcik V. (2019). Versatile Application of Nanocellulose: From Industry to Skin Tissue Engineering and Wound Healing. Nanomaterials.

[114] Sen C.K. (2019). Human Wounds and Its Burden: An Updated Compendium of Estimates. Adv. Wound Care..

[115] Mohamad N., Mohd Amin M.C.I., Pandey M., Ahmad N., Rajab N.F. (2014). Bacterial cellulose/acrylic acid hydrogel synthesized via electron beam irradiation: Accelerated burn wound healing in an animal model. Carbohydr. Polym..

[116] Shefa A.A., Sultana T., Park M.K., Lee S.Y., Gwon J.-G., Lee B.-T. (2020). Curcumin incorporation into an oxidized cellulose nanofiber-polyvinyl alcohol hydrogel system promotes wound healing. Mater. Des..

[117] Huang W., Wang Y., Huang Z., Wang X., Chen L., Zhang Y., Zhang L. (2018). On-Demand Dissolvable Self-Healing Hydrogel Based on Carboxymethyl Chitosanand Cellulose Nanocrystal for Deep Partial Thickness Burn Wound Healing. ACS Appl. Mater. Interfaces.

[118] Li Y., Liu C., Cheng X., Zhang A., Liu W., Zhang S., Jian X. (2022). Tunicate inspired gelatin-based tough hydrogel wound dressing containing twisted phthalazinone with adhesive, self-healing and antibacterial properties. Biol. Macromol..

